# Supplementary material for: No evidence that HLA genotype influences the driver mutations that occur in cancer patients
Source: Cancer Immunol Immunother. 2021 Aug 21;71(4):819–27. doi: 10.1007/s00262-021-03028-w (PMC8921139; doi:10.1007/s00262-021-03028-w)
Supplement: Supplementary file 1 — Supplementary material 1 (pdf 283 KB) [file 262_2021_3028_MOESM1_ESM.pdf]

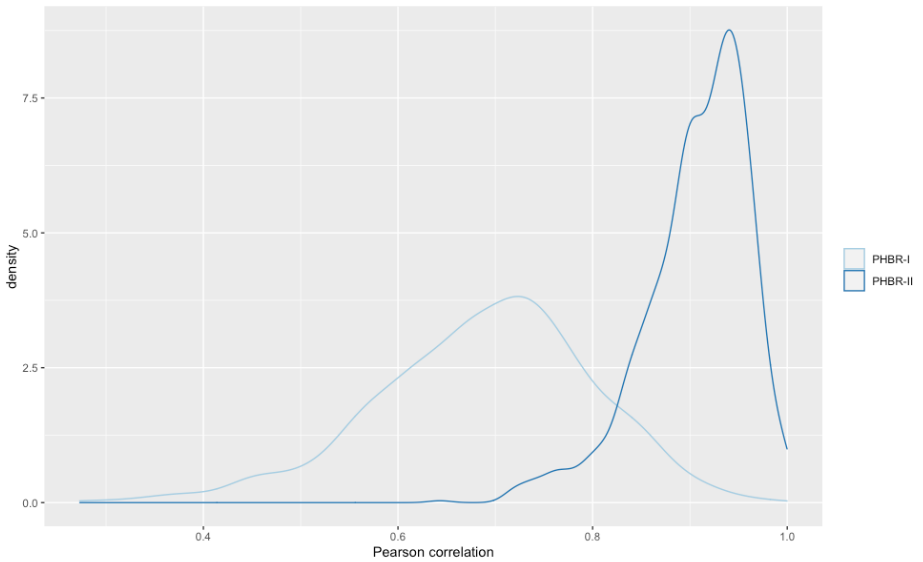

Fig S1: Density plots of the Pearson correlations of PHBR-I and PHBR-II scores between all patient pairs.

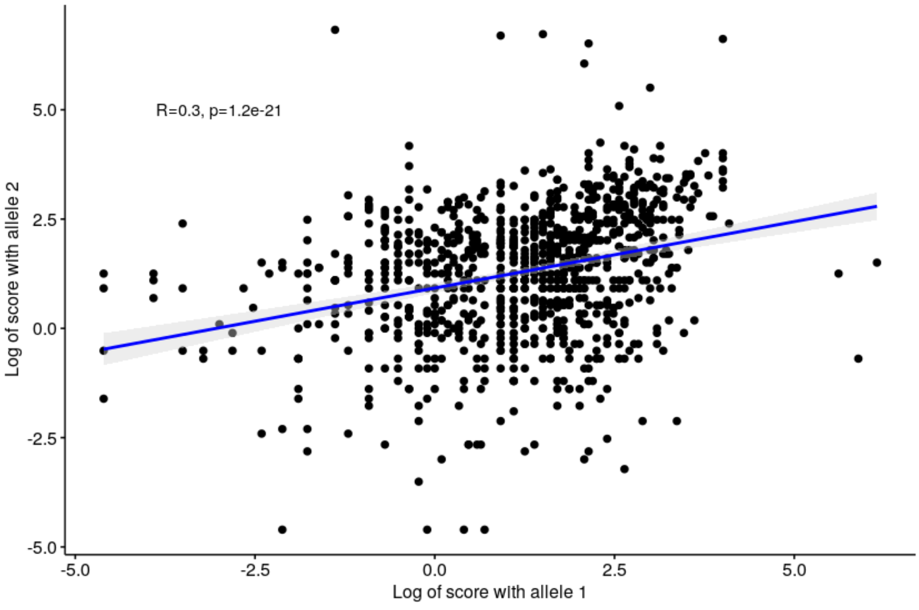

Fig S2: Correlations of PHBR-I scores from different HLA alleles

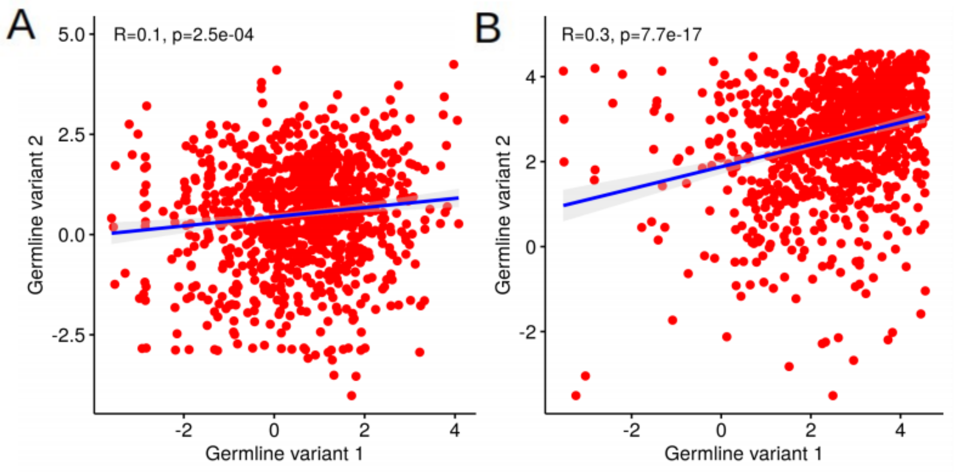

Fig. S3: Scatterplot of log PHBR-I (A) and log PHBR-II (B) scores for a random set of pairs of germline mutations on the same genes.

Table S1: Number of observations corresponding to each bin in Fig. 1C.

Bin	N (No Mutation)	N (Mutation)
3	4008601	1311
4	1550068	676
5	907929	495
6	541030	354
7	357591	273
8	348384	304
9	100837	99
10	174154	190
11	146640	176
12	100804	132
13	100793	143
14	137430	210
15	119093	195
16	64120	112
17	45795	85
18	36632	72
19	64099	133
20	36624	80
21	64085	147
22	27462	66

23	36612	92
24	27456	72
25	9151	25
26	18300	52
29	18294	58
31	9145	31
32	18288	64
34	9142	34
35	9141	35
36	9140	36
37	9139	37
39	18274	78
40	18272	80
45	9131	45
50	18252	100
62	9114	62
69	18214	138
79	9097	79
80	9096	80
88	9088	88
98	9078	98
107	18138	214
115	9061	115
137	9039	137
139	9037	139
170	9006	170
188	8988	188
215	8961	215
217	8959	217
384	8792	384
561	8615	561
